# Ubiquinol supplementation enhances peak power production in trained athletes: a double-blind, placebo controlled study

**DOI:** 10.1186/1550-2783-10-24

**Published:** 2013-04-29

**Authors:** Dietmar Alf, Michael E Schmidt, Stefan C Siebrecht

**Affiliations:** 1 Olympiastützpunkt Rhein – Ruhr, Wittekindstrasse 62, Essen 45131, Germany; 2 Biostatistics, Roentgenstr. 25, Planegg 82152, Germany; 3 Health Ingredient Consultant, Gustavstr. 36, Schwelm 58332, Germany

**Keywords:** Ubiquinol, Reduced CoQ10, Peak power output, Performance, Elite athletes

## Abstract

**Background:**

To investigate the effect of Ubiquinol supplementation on physical performance measured as maximum power output in young and healthy elite trained athletes.

**Methods:**

In this double-blind, placebo-controlled study, 100 young German well trained athletes (53 male, 47 female, age 19.9 ± 2.3 years) received either 300 mg Ubiquinol or placebo for 6 weeks. Athletes had to perform a maximum power output test and the performance in W/kg of bodyweight was measured at the 4 mmol lactate threshold on a cycling ergometer before the supplementation treatment (T1), after 3 weeks (T2) and after 6 weeks (T3) of treatment. In these 6 weeks all athletes trained individually in preparation for the Olympic Games in London 2012. The maximum power output was measured in Watt/kilogram body weight (W/kg bw).

**Results:**

Both groups, placebo and Ubiquinol, significantly increased their physical performance measured as maximum power output over the treatment period from T1 to T3. The placebo group increased from 3.64 ± 0.49 W/kg bw to 3.94 ± 0.47 W/kg bw which is an increase of +0.30 ± 0.18 W/kg bw or +8.5% (±5.7). The Ubiquinol group increased performance levels from 3.70 W/kg bw (±0.56) to 4.08 W/kg bw (±0.48) from time point T1 to T3 which is an increase of +0.38 ± 0.22 W/kg bw or +11.0% (±8.2). The absolute difference in the enhancement of the physical performance between the placebo and the Ubiquinol group of +0.08 W/kg bodyweight was significant (p < 0.03).

**Conclusions:**

This study demonstrates that daily supplementation of 300 mg Ubiquinol for 6 weeks significantly enhanced physical performance measured as maximum power output by +0.08 W/kg bw (+2.5%) versus placebo in young healthy trained German Olympic athletes. While adherence to a training regimen itself resulted in an improvement in peak power output, as observed by improvement in placebo, the effect of Ubiquinol supplementation significantly enhanced peak power production in comparison to placebo.

## Background

Coenzyme Q10 (CoQ10) is synthesized in the human organism and is a fat soluble, vitamin-like substance which can exist as Ubiquinone (oxidized CoQ10) or as Ubiquinol (the unoxidized, reduced form). It plays various roles in the energy production of the muscles’ cells. The concentration of the coenzyme in the tissue can decline, and thus be suboptimal, as a consequence of different pathological changes. In addition, additional factors that can negatively influence CoQ10 levels include intensive training and aging. Long lasting and intensive efforts by sport training likewise contribute to this reduction. Some existing studies have already shown that CoQ10 can mitigate muscle damage after high level training [1]. Previous studies have been conducted utilizing differing dosage levels of CoQ10 and have shown conflicting results. Coenzyme Q10 was previously considered to be an ineffective substance for athletes, as past studies with CoQ10 did not give consistent results. This may have been caused by the study design or by an insufficient dosage of CoQ10.

### Energy production in mitochondria via CoQ10 and Ubiquinol

CoQ10 is an integral component of the mitochondrial oxidative phosphorylation system, where it serves as an essential carrier of reducing equivalents in electron transport. Oxidative phosphorylation harnesses energy from nutrients to produce ATP, the energy in each of our cells and all of our life processes. CoQ10 is critical for the synthesis of ATP, as 96% of all aerobically produced energy involves CoQ10. Though it is endogenously synthesized, a small amount of CoQ10 is always degraded and thus must be replenished from dietary sources. Organs like the heart and muscles, which require consistent and robust bioenergetics, depend on a sufficient supply of CoQ10 and produce less energy and strength if they are deficient in CoQ10.

### Antioxidant function of CoQ10 and Ubiquinol in cell membranes

CoQ10 is the most important lipid soluble antioxidant in the body along with vitamin E. They are structurally linked to one another and both are part of the cell membranes which they protect from deleterious radicals. In fact, CoQ10 in the Ubiquinol form is depleted before vitamin E, as it reacts first with radicals and is destroyed by them [2]. CoQ10 in the Ubiquinol form is a potent antioxidant that has the capacity to protect Vitamin E, and also helps to regenerate depleted vitamin E and Vitamin C. Oxidized CoQ10 (ordinary CoQ10) must first be converted to the Ubiquinol form in order to exert this antioxidant effect. CoQ10 should not be compared with the multitude of water soluble antioxidants, which move freely in the blood and have a rather non-specific effect. Along with vitamin E, CoQ10 has the special task of protecting the very sensitive cell membranes and this gives it a unique position amongst all antioxidants.

Studies have shown that the reduced form of CoQ10 known as Ubiquinol is 6–10 times more bioavailable than oxidized CoQ10 [3,4]. Plasma levels of 6–8 μg/ml plasma can be achieved in humans with 300 mg Ubiquinol [3]. With 450 – 600 mg Ubiquinol, CoQ10 plasma levels of 8–10 μg/ml plasma can be achieved [5]. Studies are currently underway, also with trained elite athletes in Germany, to determine whether athletes in particular can benefit from such elevated CoQ10 plasma levels.

The optimal plasma level for athletes is not known to date. It appears that athletes need more CoQ10 due to their higher metabolic requirement, and CoQ10 supplements may benefit them by increasing their plasma and muscular CoQ10 levels. The necessary and effective dosages for athletes remain unknown yet. A typical plasma level of 1 μg CoQ10 per milliliter of plasma may not be enough to optimize physical performance. Previous studies have shown that only athletes with a CoQ10 Plasma level greater than >2.5 mg/L (=2,5 μg/ml) or more showed an increase in physical performance. Athletes want to get the highest possible CoQ10 plasma levels of greater than >3.5 mg/L (=3,5 μg/ml) [6].

Despite *de novo* synthesis of CoQ10, it appears to be lost during the sustained exertion required in sports training. Trained athletes often have lower CoQ10 plasma levels than untrained people [7]. Heavy training and exercise leads to a decrease in plasma levels of athletes [8]. The athletes had lower plasma levels during periods of heavy training than in training free periods [9]. This may be caused by different mechanisms. Athletes appear to have a higher metabolic requirement of CoQ10, which is not compensated by normal food intake and biosynthesis in the body. Highly trained athletes can therefore exhibit lower CoQ10 levels in tissue and blood, and this can limit their performance. So it is especially important for athletes to monitor their CoQ10 plasma level and to supplement their CoQ10 as necessary. To date, there is no recommended CoQ10 plasma level for athletes. But the latest studies show a link between the CoQ10 plasma level and performance capacity: the higher the CoQ10 plasma level, the higher the performance capacity. Higher CoQ10 plasma levels may translate into higher CoQ10 levels in muscles and liver. Kon et al. [10] demonstrated that CoQ10 supplementation increased total CoQ10 concentration significantly in slow-twitch muscles (soleus and gastrocnemius deep portion) and liver. Additionally, plasma creatine kinase was significantly decreased after exercise by CoQ10 supplementation as opposed to placebo. Coenzyme CoQ10 deficiency in athletes could be triggered by:

• Increased consumption and increased requirement for CoQ10 due to sustained, heavy physical exertion

• Reduced CoQ10 uptake due to vegetarian diet

• Limited CoQ10 biosynthesis due to deficiencies of nutrients like selenium, vitamin B6, magnesium etc.

• Intake of high doses of vitamin E inhibits CoQ10 uptake from food and lowers the CoQ10 plasma level

• Statin therapies limit CoQ10 biosynthesis and deplete the CoQ10 plasma level

Normally, training can increase the number of mitochondria in heart and muscles. The mitochondria are rich in CoQ10 and therefore training also increases the CoQ10 content in heart and muscle [11]. Training also increases the biosynthesis of CoQ10 and therefore there is also a higher requirement for ingredients that are needed for the CoQ10 biosynthesis. On the other hand, the mitochondria normally do not reach the CoQ10 saturation level [12]. This practically means that at the actual concentrations of CoQ10 in these membranes the velocity of the respiratory complexes is not the maximal one. There is still capacity to increase the CoQ10 content in the mitochondria, and this could explain the increase of maximal oxygen uptake (VO2-max) by CoQ10 supplementation [9]. Heavy physical training leads to a decrease in plasma CoQ10. Plasma CoQ10 is inversely correlated to the intensity of training or exercise.

The muscle CoQ10 content is linear dependent on the content of Type I, oxidative muscle fibers [13]. In a study by Fiorella and Bargossi [14], the CoQ10 Plasma level increased less after supplementation when the athletes exercised heavily. It seems that the CoQ10 in the plasma is immediately absorbed by the exercising muscle. Exercise may stimulate the muscular uptake of CoQ10 from the plasma.

### CoQ10 dosage for athletes

In animal models, administration of CoQ10 has shown an increase in CoQ10 concentrations in organs, in particular the heart and muscle. In these studies it was also shown that CoQ10 supplementation also increased Vitamin E content in heart muscle and liver [15].

In humans, a dosage of 120 mg CoQ10 given to athletes was unable to increase the muscle CoQ10 content [16]. To increase the human muscle CoQ10 content, it is necessary to increase the CoQ10 plasma to a greater extent over a longer period of time, so that the muscle tissues have enough time to absorb the CoQ10 from the plasma. Higher dosages of 200–300 mg CoQ10 or more of Ubiquinol per day over a 4–12 week period is needed to increase muscle CoQ10 content. In one trial, 200 mg CoQ10 supplementation for 14 days lead to a trend of in increased muscle CoQ10 content [17]. Based on these observations, 100 mg CoQ10 per day for athletes may be insufficient to achieve any enhancement in performance. Indeed, earlier studies were likely unsuccessful because of inadequate dosing, resulting in suboptimal CoQ10 plasma levels. In an earlier Italian study, a dosage of 100 mg CoQ10 per day only increased the plasma level to a value of 1.34 μg/ml [18], which is too low to achieve any effects for athletes.

In a later Italian study the same 100 mg dose raised the CoQ10 plasma level to 2.23 μg/ml.

After 2 months of CoQ10 supplementation, greater exertion was required to induce exhaustion and overall performance improved. Another study found the dose of 100 mg CoQ10 exerted no effect, but a 300 mg dosage of CoQ10 and raising plasma level to 3.29 μg/ml significantly increased endurance and protected against exhaustion in a maximum speed test on the ergometer [10]. In a crossover study of 15 cyclists in which each participant received both 300 mg of CoQ10 and placebo, each for four weeks in random order, a moderate to strong correlation between the significant increase in total blood CoQ10 and total workload was observed [19]. Given the small sample size and the crossover study design that administered CoQ10 at different phases of the athletes’ overall training regimen, the correlation between total blood CoQ10 and performance improvement suggests that a sufficiently powered study with a traditional placebo-controlled design where the 300 mg dosage was administered for at least four weeks or more could evaluate whether CoQ10 affects performance output.

Based on the available data, it appears that the CoQ10 dosages in earlier studies were insufficient to achieve any significant positive results for athletes. Clinical studies with athletes are increasingly proving positive effects for a dosage of 300 mg CoQ10 or CoQ10 plasma levels >3.3 μg/ml. With Ubiquinol, the reduced form of CoQ10, higher CoQ10 plasma levels can be achieved with lower dosages than with oxidized CoQ10 which might be metabolically superior. This study extends the findings of previous studies by enrolling a study population with greater statistical power and administering either CoQ10 at 300 mg daily or placebo for six weeks to elite athletes in a variety of sports at a similar stage in their training regimen in preparation for the Olympic Games of 2012.

## Methods

One hundred subjects (gender of the athletes: 53 males and 47 females) were recruited among the young German athletes training regularly at the Olympic Training Camp Rhein-Ruhr in Essen, many of whom are directly competing at the Olympic Games 2012 in London. No monitoring or control of diet (e.g., fasting) was imposed on study participants to mimic the circumstances under which supplements are typically ingested by athletes, both elite and recreational. This investigation sought to compare the performance effect of 50 athletes on Ubiquinol supplementation versus 50 other athletes who received placebo capsules.

All athletes received 5 brown colored liquid filled hard gelatin capsules every day. These capsules contained either lactose in medium chain triglycerides (MCT) Oil (placebo group) or 60 mg Ubiquinol in MCT oil (KanekaQH) per liquid filled hard gelatin capsules capsule. The liquid filled hard gelatin capsules were produced by Capsugel (Colmar, France). The athletes came from the training pool of the following respective sports: canoe, rowing, swimming, hockey, golf, track and field.

At study entry the athletes were randomly assigned to receive liquid filled hard-gelatin capsules containing Ubiquinol or placebo. The average age of the tested people was 19.2 years (±2.3 years). The average height was 181 cm (±10.5 cm) and the average weight 78 kg (±19.7 kg).

The performance is expressed in Watts per kilogram body weight (W/kg bw), and measured at the beginning of the Ubiquinol supplementation and of the placebo group, after 3 weeks and after 6 weeks. Lactate levels were checked in parallel with blood samples. The tests were performed on the IAS 150 from the company Ergoline, which measures Watt performance. Based on performance time, the work load per kg of body weight was calculated (W/kg bw).

Physical performance is usually measured by a gradual, continuous or intermittent shaped rising stress test during spirometry determined on a bicycle or treadmill [20-22].

### Statistical analysis

The data were derived from a placebo-controlled, randomized, two-arm study which was initiated to investigate the effect of Ubiquinol in improving the physical fitness of trained athletes (a total of 100 young healthy athletes, ratio of control to experimental subjects = 1:1, n = 50 in experimental and n = 50 in control group, respectively). The physical performance of the athletes was measured at three different time points (T1, T2, T3) in watts per kilogram of body weight (W/kg bw). The primary endpoint of the study was defined as the difference of the mean fitness increase of both groups measured from time point T1 to time point T3.

After determining the individual fitness increase from time point T1 to time point T3 the significance of the difference of the group means (experimental: mean = 0.38, standard deviation = 0.22; control: mean = 0.24, standard deviation = 0.34) was calculated using a Student’s *t*-test for independent samples and pooled variances. The test statistic revealed significant differences between the control and experimental groups with a p-value of 0.018 on an error level of α = 0.05.

### Statistical methods

The variables set included the fitness measurements at the time points T1, T2, and T3 as well as the subject identification number. In the univariate analysis, line graphs depict the individual’s fitness level at different time points throughout the study and the fitness means of both groups including one standard deviation. Histograms are used for screening of outliers, checking normality, or suggesting another parametric shape for the distribution. The two-sided Student’s *t*-test for independent samples and pooled variances was applied for testing the statistical significance of the difference between the mean fitness increases of the two groups based on log-transformed values. The Fisher’s F-test was used to compare two variances. The goodness of fit of the sample to a normal distribution was assessed using the Kolmogorov-Smirnov test and Q-Q plot (not shown). Finally, a linear mixed-effects model was fitted simultaneously to all measurements of both groups. The statistical testing’s were conducted using an exploratory approach, the maximum type I error probability associated with all statistical tests in the analyses is 0.05. The biometric analyses were performed with the statistical programming environment GNU R, version 2.14.0, and the post hoc power analysis was computed using PS Power and Sample Size Calculations, version 3.0.

## Results and discussion

### Statistical results

#### Univariate analysis

**Figure 1:**
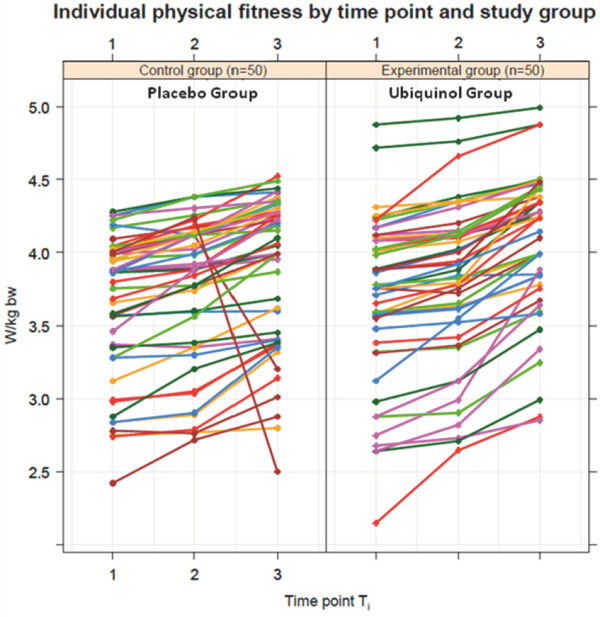
**Individual physical fitness by time point and study group.** Individual performance output measured in W/kg bw at time points T1, T2 and T3, stratified by placebo group (Control group) and Ubiquinol group (Experimental group).

The individual fitness levels measured in Watt/kg bodyweight at time points T1, T2 and T3, and stratified by study group, are illustrated in Figure 1. As one can see from the graph, two athletes of the control group show normal increases of their values at time point T2, but are followed by implausible deep declines at time point T3. The drop in physical performance was due to an infection, therefore the two individuals are considered to be protocol non-compliers, and the corresponding records are dropped from computations, otherwise these two data would have had a quite negative impact of the performance of the placebo group and would have created a wrong and too positive difference in performance towards the Ubiquinol supplement group. Thus, in total n = 50 athletes of the experimental group and n = 48 athletes of the control group finally remained for further analysis.

**Figure 2:**
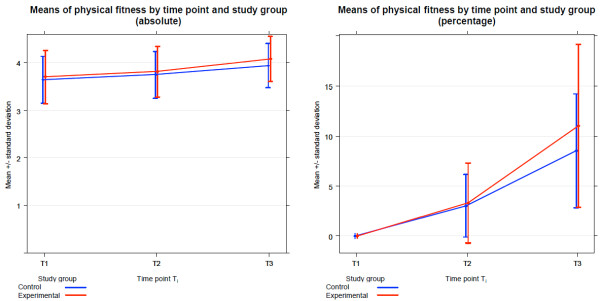
**Mean Measured fitness by time point and study group.** Progress of fitness (absolute values in W/kg bw and percentage values) at time points T1, T2 and T3 plotted as means and one standard deviation, stratified by study group.

The arithmetic means of the power output measurements increased from 3.70 W/kg bodyweight (±0.56) at time point T1 to 4.08 W/kg bodyweight (±0.48) at time point T3 in the experimental group and from 3.64 W/kg bw (± 0.49) to 3.94 W/kg bw (±0.47) in the control group, respectively (Figure 2). This corresponds to mean differences between the time points T1 and T3 of 0.38 W/kg bodyweight (±0.22) in the experimental group and of 0.30 W/kg bodyweight (±0.18) in the control group. Accordingly, the mean percentage increases at time point T3 calculated with respect to time point T1 are 11.0% (±8.2) in the experimental (ubiquinol) group and 8.5% (±5.7) in the control (placebo) group. For both study groups, the calculated statistical parameters are summed up in Table 1.

**Table 1:** Summary Statistics

Parameter	**Experimental group**						
	N	Mean	95% CI	Std	Min	Med	Max
T1	50	3.70	3.54-3.86	0.56	2.14	3.77	4.88
T2	50	3.81	3.66-3.96	0.53	2.65	3.90	4.92
T3	50	4.08	3.94-4.21	0.48	2.85	4.24	4.99
Diff. abs. T1-T3	50	0.38	0.32-0.44	0.22	0.07	0.34	1.13
Diff. perc. T1-T3	50	11.03	8.71-13.55	8.16	1.62	8.58	41.09
Parameter	**Control group**						
	N	Mean	95% CI	Std	Min	Med	Max
T1	48	3.64	3.50-3.78	0.49	2.42	3.86	4.28
T2	48	3.75	3.60-3.89	0.49	2.72	3.89	4.38
T3	48	3.94	3.80-4.07	0.47	2.80	4.08	4.52
Diff. abs. T1-T3	48	0.30	0.25-0.35	0.18	0.03	0.28	0.76
Diff. perc. T1-T3	48	8.54	6.89-10.20	5.70	0.84	7.20	21.97

Summary statistics of the measured fitness in W/kg bw at time points T1, T2 and T3, and the fitness increases expressed as absolute (Diff. abs.) and percentage values (Diff. perc.) including number of probands (n), arithmetic mean (Mean), 95% confidence limits of mean (95% CI), standard deviation (Std), minimum (Min), median (Med) and maximum (Max), stratified by study group.

Before performing tests of significance, a log-transformation of the computed fitness differences between time point T1 and time point T3 was applied to make the variable’s distribution closer to normal. Hence, no significant deviation from the normal distribution could be detected (Kolmogorov-Smirnov test: experimental p = 0.995, control p = 0.381), and the variances were homogenous (F-test: p = 0.112), which is considered to be a precondition for performing a *t*-test. The *t*-test revealed a significant difference of the mean fitness increases between experimental and control groups (p = 0.03).

#### Multivariate analysis

A linear mixed effects model was used to analyze the resulting figures, controlling for time and group effects. The model includes the fitness values in Watt per kg bodyweight on the original scale as response variable, with repeated measurements at time points T1, T2, T3 and study group as fixed factors. The number of the athletes was added to the model as a random variable to accomplish an individual level estimation. Time point T1 and the control group were used as reference category. The parameter estimates for the predictor variables were obtained using restricted maximum likelihood technique with stepwise forward selection. The results of the main effect analysis indicate a highly significant influence of training time regarding progress of physical fitness (T2 and T3 p < 0.001). Furthermore, the interaction between study group and time point T3 is noticeably significant (p = 0.010). Thus, multivariate analysis also demonstrates that both study groups experienced a substantial increase in physical fitness. However, this training effect is significantly more apparent in the experimental group (Ubiquinol supplementation) than in the control (placebo) group.

## Discussion

Among these 100 young and healthy elite German Olympic athletes, a continuous increase of physical fitness was observed in the Ubiquinol supplemented group as well as in the control group during the study course, expressed in absolute values or in percentage units. This effect is attributed to the individual physical training program of each athlete, and matches the expectation. However, the objective of the study was to investigate to what extent the effect of physical training can be positively influenced by additional intake of 300 mg Ubiquinol daily for six weeks as a dietary supplement. Based on the available data, the results of the study suggest that Ubiquinol may have positively impacted the observed elevated level of training success, a fact that was statistically significant for absolute differences and in multivariate analysis but slightly missed the significance level using percentage values. However, the numerical difference between experimental and control groups regarding the effect of Ubiquinol might be regarded as relatively small, but this can make a very significant difference for elite athletes.

Elite athletes are training on such a high level that performance enhancements often fail to impart any additional ergogenic benefit. In other studies for example it was shown that caffeine can increase mean power output in a similar range as we found here for Ubiquinol. In one double blind, randomized crossover study, a supplementation with 6 mg or 9 mg caffeine per kg body weight increased performance by +2.7% (+0.4 to +5.0%) and decreased performance time in rowers 2000-m distance by −1.2% (−0.4 to −1.9%) vs placebo [23]. The used dosage in this study is quite high and bears some health risks especially for the cardiovascular system. Both doses of caffeine had a similar ergogenic effect relative to placebo. So there is no benefit of consuming more caffeine, but the negative side effects of caffeine are increasing. The magnitude of the performance enhancement is already achieved by 3 mg caffeine per kg bodyweight and was found to be around +0.4 to +5% in different studies [24,25].

Though caffeine generally accepted as an ergogenic aid, it was on the official doping list for decades and banned since 2004. Because high caffeine consumption may cause serious side effects especially for athletes, the World Anti-Doping Agency is considering banning caffeine again to avoid potential health risks for athletes. Nutrients such as Ubiquinol are a safe and healthy alternative to caffeine as on one hand it supports and increases physical performance of the athletes in a similar range like caffeine and secondly is also beneficial for the health of the athletes, especially for the heart. Additionally, Ubiquinol may in particular benefit the antioxidant status of athletes which often compromised by the elevated presence of reactive oxygen species.

The results of the test statistics have been advantageously affected by the small variability of increase of physical fitness among the two study groups despite the range of intensity of physical activity inherent to the sports in which each athlete was training (e.g., golf vs. track and field). The plot of the individual performance output (Figure 1) suggests that individuals exist in the experimental group who benefitted more from an Ubiquinol supplement compared to others. Two participants of the control group were initially excluded from the analysis. If these two participants had remained in the study, the effect differences between the two study groups would have been larger, resulting in considerably higher statistical significance. Further insight could be provided, if the enhancement of performance output could be correlated with other biological parameters, e.g. the individual Ubiquinol plasma levels of the athletes. Future studies might benefit from being designed to provide CoQ10 at individualized doses that achieve a consistent range of plasma Ubiquinol concentration. Physical training leads to an increase in muscle mass and also to an increase in mitochondria containing Q10. Increased demand for Q10 by muscle could explain why plasma Ubiquinol levels have been observed to decrease in trained athletes [6,7].

Certain data measured in previous studies (e.g., plasma Ubiquinol concentration and oxidative stress) were not collected in this study due to lack of available funds to perform these relatively expensive assays multiple times in a study population of 100. Another consideration in the choice not to measure oxidative stress was that its link with physical performance has not been established. The goal of this study was to focus on CoQ10’s energetic effects and not on its antioxidant properties. Another difference between this study and some previous studies is the lack of control or monitoring of dietary intake; however, Q10 intake via food consumption ranges between 5–10 mg per day, a level that is insignificant relatively to the administered dose of 300 mg per day. So, while there may have been variance among study participants with regards to diet, oxidative stress, and plasma concentrations of Ubiquinol, such variances were insufficient to negate the statistical significance of the findings on CoQ10’s effects on physical performance as reported here.

In this study, CoQ10 supplementation resulted in increased short term maximum performance, which implies anaerobic output, perhaps via an increase in ATP and creatinine phosphate synthesis. An alternative explanation is that CoQ10 supplementation could work via a direct increase in muscular Q10 levels, suggesting that aerobic energy conversion might be improved by inhibiting ammonia production from AMP. When ATP levels decrease during exercise, 2 ADP are converted into ATP and AMP. Higher mitochondria activity produces more continuous ATP and a higher level on Ubiquinol in the mitochondria contributes to increased ATP synthesis. Such mechanisms are consistent with the observation of improved performance with CoQ10 supplementation over a study population that included both endurance and strength athletes.

Older athletes and “weekend warriors” might profit even more from CoQ10 supplementation than young, well-trained athletes. Aging reduces the number of mitochondria and the level of Q10 in all tissues decreases with age. Increasing the Q10 content of remaining mitochondria might at least partly compensate for the lower number of mitochondria. Untrained athletes’ muscles are not as adapted to changing energy needs during exercise as are those of elite athletes. Other supplements have elicited stronger effects in increasing physical performance in recreational athletes and CoQ10 might be another such example.

## Conclusions

This study demonstrates that daily supplementation of 300 mg Ubiquinol for 6 weeks significantly enhanced physical performance measured as maximum power output by +0.08 W/kg bw (+2.5%) versus placebo in young healthy trained German Olympic athletes. While adherence to a training regimen itself resulted in an improvement in peak power output, as observed by improvement in the placebo group, the effect of Ubiquinol supplementation significantly enhanced peak power production in comparison to placebo.

## Abbreviations

ATP: Adenosine triphosphate; CoQ10: Coenzyme Q10; kg: Kilogram; l: Liter; μg: Microgram; mg: Milligram; mL: Milliliter; T1: Timepoint prior to supplementation treatment (either experimental or control); T2: Three weeks after initiation of supplementation treatment; T3: Six weeks after initiation of supplementation treatment; VO2-max: Maximal oxygen uptake or maximal aerobic capacity; W/kg bw: Watt/kilogram body weight.

## Competing interests

The study was funded by the companies and Capsugel an Kaneka Pharma Europe.

## Authors’ contributions

DA carried out the study and collected the data, MS made all the statistical calculations, SS participated in the sequence alignment and drafted the manuscript. All authors read and approved the final manuscript.
